# A preliminary phylogeny of the South African Lentulidae

**DOI:** 10.1186/s41065-015-0005-6

**Published:** 2016-01-15

**Authors:** Daniela Matenaar, Linda Bröder, Axel Hochkirch

**Affiliations:** 1 Department of Biogeography, Trier University, D-54286 Trier, Germany; 2 State Museum of Natural History Stuttgart, Department of Entomology, D-70191 Stuttgart, Germany

**Keywords:** Biodiversity hotspot, Cape Floristic Region, Invertebrates, Orthoptera, Taxonomy

## Abstract

**Background:**

The grasshopper family Lentulidae is endemic to eastern and southern Africa, with its center of diversity situated in South Africa, the highest diversity being found in the Cape Floristic Region, which is one of the global biodiversity hotspots. The family consists of 35 genera sorted in two subfamilies. This study provides first insights into the phylogeny of Lentulidae. Two mitochondrial genes (12S and NDS) were sequenced and the phylogeny was inferred through Maximum Likelihood and Bayesian Inference.

**Results:**

Our results indicate that the current classification into the subfamilies Lentulinae and Shelforditinae may be incorrect as *Uvarovidium, Leatettix* (Shelforditinae) and *Devylderia* (Lentulinae) clustered together in one main clade, while *Betiscoides, Basutacris* and *Gymnidium* (all Lentulinae) formed the second main clade. The genera *Uvarovidium* and *Leatettix*, which had been assigned to the Acrididae (subfamily Hemiacridinae) in the past, grouped within the Lentulidae, confirming their current assignment to this family. The East African *Usambilla* group is likely to represent a sister clade to the south African *Lentula* and *Eremidium*. Diversification patterns in the genus *Devylderia* and *Betiscoides* suggest a higher number of species than currently known.

**Conclusions:**

Our phylogeny is not in line with the current systematics of Lentulidae, suggesting that a broader sampling and a study of the genitalia would be useful to clarify the taxonomy. Furthermore, some genera (particularly *Betiscoides* and *Devylderia*) are in need of taxonomic revision, as the number of species within these genera is likely to be higher than the current taxonomy suggests.

**Electronic supplementary material:**

The online version of this article (doi:10.1186/s41065-015-0005-6) contains supplementary material, which is available to authorized users.

## Background

The Cape Floristic Region in South Africa is one of the global biodiversity hotspots [1]. Numerous studies have dealt with the enormous plant diversity and its origin and a number of phylogenetic studies on certain plant families aimed at unravelling the drivers for diversification in this region [2–4]. The fauna of the Cape Floristic Region, though not less unique, has not been investigated as intensely as the flora, especially research on invertebrate diversity remains still scarce [5]. Recent evidence suggests that invertebrate diversity and endemism in the Cape Floristic Region might be comparable to the pattern found in plants [5, 6]. The Cape Floristic Region also maintains a variety of endemic families, subfamilies and genera, suggesting that it provided refugia also over a longer time-span. One of these subfamilies endemic to the Cape Floristic Region is the subfamily Shelforditinae Ritchie, 1982 within the family Lentulidae Dirsh, 1956. Within the family Lentulidae as a whole, the majority of taxa (70 %) are also endemic to South Africa [7], but some genera occur in East Africa, particularly the *Usambilla* group, which shows a radiation in the East African mountain systems [8].

All Lentulidae are completely wingless, and therefore expected to show high levels of genetic differentiation at a small geographic scale, as they have a low mobility and are often adapted to certain vegetation structures. For example, the Lentulidae genus *Betiscoides* Sjöstedt, 1923 is adapted to Restionaceae, which also show a high level of differentiation in the Cape Floristic Region [9]. Studies on the phylogenetics of grasshoppers from the Cape Floristic Region are lacking so far. However, several recent taxonomic studies suggest a high level of differentiation of flightless grasshopper species in the Cape Floristic Region with the identification of tens of hitherto undescribed species [10, 11]. Given the enormous number of unidentified species in times of increasing efforts to halt the loss of biodiversity, there have been approaches to record and assess biodiversity parameters also on higher taxonomic level [12]. Thus, it is of increasing interest to investigate the systematic relationships of endemic taxa to understand the importance of biodiversity hotspots.

The aim of our study was to investigate and reconstruct the phylogenetic relationships among genera within the family Lentulidae. We were particularly interested in the systematic position of the genera *Leatettix* Dirsh, 1956 and *Uvarovidium* Dirsh, 1956, which are currently assigned to the subfamily Shelforditinae. Furthermore, we aimed to clarify the phylogenetic relationships of the East African Usambilla group (here represented by the genera *Usambilla* Sjöstedt, 1910 and *Rhainopomma* Jago, 1981) with respect to the South African Lentulidae. We present the results of a first phylogenetic study on Lentulidae based upon two mitochondrial genes (12S rRNA and NDS – a fragment containing parts of the 16S rRNA, t-Leu and ND1), covering ten genera from both subfamilies, in order to provide new insights in evolutionary history of this family.

## Results

The complete alignment contained 808–823 bp. The 12S fragment was between 318 and 327 bp (when including the outgroups between 316 and 327 bp) and NDS (consisting of a fragment of 16S, t-Leu and ND1) between 490 and 497 bp long (with outgroup: 487–497). NDS had more variable sites (254) compared to 12S with 178 variable sites (see Table 1), but the percentage of variable sites was slightly larger in 12S (54 % vs. 51 %). The highest genetic distance (p-Distance for the 12S data set) between genera within Lentulidae was detected between *Usambilla* and *Uvarovidium* with *p* = 0.224, whereas the lowest was found between *Basutacris* and *Betiscoides* (*p* = 0.057, Table 2).

**Table 1:** Overview on gene specific parameters for genes 12S and NDS (16S + t-Leu + ND1) of the combined data set

Sequence	Number of			Nucleotide frequency [%]			
	bp	indels	variable positions	T	C	A	G
12S	327	8	178 (54.4 %)	41.4	10.6	31.3	16.6
NDS	497	8	254 (51.1 %)	45.6	10.4	30.0	14.0

**Table 2:** Genetic distances (p-Distance) between the analyzed genera of Lentulidae based upon the 12S data set

	*Devylderia*	*Betiscoides*	*Leatettix*	*Uvarovidium*	*Gymnidium*	*Basutacris*	*Usambilla*	*Lentula*	*Eremidium*
*Betiscoides*	0.115								
*Leatettix*	0.129	0.142							
*Uvarovidium*	0.124	0.147	0.104						
*Gymnidium*	0.118	0.078	0.151	0.155					
*Basutacris*	0.116	0.057	0.137	0.142	0.069				
*Usambilla*	0.193	0.176	0.217	0.224	0.172	0.172			
*Lentula*	0.169	0.140	0.169	0.170	0.153	0.151	0.204		
*Eremidium*	0.136	0.105	0.152	0.143	0.113	0.114	0.182	0.137	
*Rhainopomma*	0.172	0.135	0.192	0.214	0.135	0.144	0.092	0.165	0.137

**Fig. 1:**
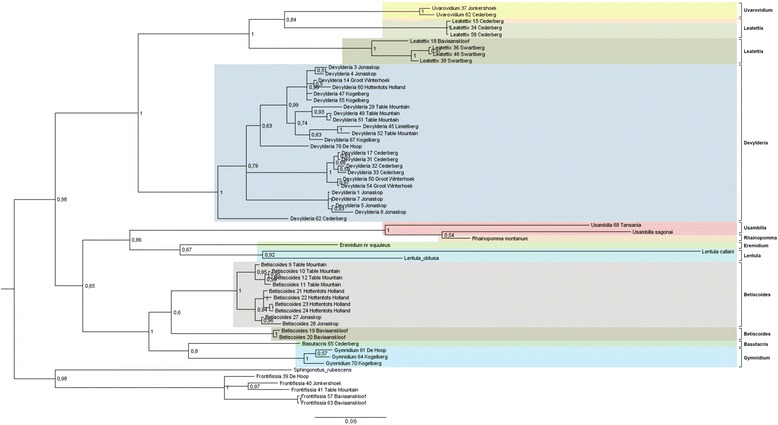
Shows the inferred phylogeny of the analyzed genera belonging to Lentulidae. Consensus tree of the Bayesian Inference for the genes 12S and NDS; 824 bp in total, 20 million generations with sample frequency of 2000. *Frontifissia* and *Sphingonotus* were defined as outgroups

The phylogenetic reconstruction showed similar results for both methods, i.e. Maximum Likelihood and Bayesian Inference. Thus, only the results derived from the Bayesian Inference are presented and discussed here in detail. The Maximum Likelihood tree is provided in Additional file 1: Figure S1. The consensus tree from the Bayesian analysis showed a monophyly of the Lentulidae with respect to the chosen outgroups *Sphingonotus rubescens* and *Frontifissia elegans* and *F. laevata* (Fig. 1). Within the family Lentulidae, a clear basal split between two main clades was revealed, which was supported by a BPP value of 0.98. The first main clade consisted of the genera *Gymnidium*, *Basutacris*, *Betiscoides, Lentula, Eremidium, Rhainopomma* and *Usambilla.* Specimens collected in the same region clustered together, e.g. Table Mountain (*Betiscoides*), Hottentots Holland (*Betiscoides*), Jonaskop (*Betiscoides*), Baviaanskloof (*Betiscoides*), and Kogelberg (*Gymnidium*). The East African *Usambilla* group (genera *Usambilla* and *Rhainopomma*) formed a monophyletic group, related to the South African genera *Eremidium* and *Lentula*.

The second main clade, consisting of the genera *Devylderia*, *Uvarovidium* and *Leatettix*, revealed a rather distinct pattern regarding their geographical distribution. *Leatettix* specimens from Cederberg clustered in a single clade, strongly separated from the clades in Swartberg and Baviaanskloof, as a sister clade to *Uvarovidium*. However, this relationship was supported by a BPP value of only 0.84. *Devylderia* formed four subclades, one including individuals from Jonaskop, one from Cederberg and Groot Winterhoek, one represented in Jonaskop, Table Mountain, Limietberg, Hottentots Holland, Groot Winterhoek and De Hoop, and the fourth subclade included only one specimen from Cederberg.

## Discussion

The results provide first insights into the phylogeny of the family Lentulidae. The clear split between the two main clades was highly supported. However, this split does not reflect the current subdivision into the two subfamilies Lentulinae and Shelforditinae. Even though the two Shelforditinae genera *Leatettix* and *Uvarovidium* clustered as a monophylum, they showed a clear sister group relationship to the genus *Devylderia* (Lentulinae). This could mean that either *Devylderia* belongs to Shelforditinae as well or that the current taxonomy within the complete family needs to be revised. A broader taxon sampling across all described lentulid genera would provide more precise information on the systematic relationships of the genera and clarify the assignments to the respective subfamilies. The East African *Usambilla* group is likely to represent a sister clade to *Lentula* and *Eremidium*, confirming their systematic position within the subfamily Lentulinae. This also suggests that the Lentulidae as a whole originated in South Africa and started to diversify here, before one group (the ancestor of the *Usambilla* group) spread to the north to radiate within the East African Mountain systems.

Although our sampling only covers a small fraction of the Lentulidae genera, a strong genetic differentiation between and within genera becomes already evident. In some cases, our phylogeny does not reflect the current taxonomy. This is the case for the genus *Leatettix*, which does not represent a monophylum, but a paraphyletic group. Otte [11] mentioned in his revision of *Leatettix* that further division of *Leatettix* species into different genera might be necessary. As *Leatettix emota* from Cederberg are closer related to *U. peninsulare* than to *Leatettix moraki* from Swartberg and Baviaanskloof, his suggestion seems to be supported by our findings. Further morphological and genetic research is required to clarify these findings.

Recent revisions of several lentulid genera from South Africa showed that the number of Lentulidae species is much higher than currently known. In total, 44 new species and even three new genera have recently been described (*Armstrongium*, *Tanquata*, *Tsautettix,* [13]). The center of species richness of Lentulidae is in South Africa with 103 of the 146 described species occurring in the Cape Floristic Region [7]. Recent studies show that insect species diversity in the Cape Floristic Region is generally much higher than current taxonomy suggests [10, 14]. This seems to be particularly true for the genus *Betiscoides* (Matenaar et al. unpubl.), which is also confirmed in our study. Although this genus represents a monophylum, specimens from different localities showed a high genetic differentiation. All specimens used in our study would morphologically be assigned to *Betiscoides meridionalis*. However, the results indicate four clades of this species with high genetic differentiation, suggesting the existence of cryptic species (which is confirmed by first morphological inspection).

The reasons for the unique diversity in the Cape Floristic Region and potential drivers of differentiation and speciation have been discussed before [2, 3, 15]. Concerning insect diversity, there is a consensus about the long-term isolation of populations starting in the early Miocene [16–18]. The Cape Floristic Region experienced climatic changes during late Mio- and Pliocene and repeated orographic changes through the uplift of the Cape Fold Belt as well as oceanic regression. These changing environmental conditions probably triggered and influenced dispersal as well as survival of insect taxa in refugia. Coastal regions and mountains of the Cape Floristic Region probably functioned as refugia during unfavorable periods, whereas ocean regression repeatedly enabled taxa to disperse into lowland habitats and the interior of the Cape Floristic Region [19]. Climatic stability in mountain ranges throughout the Pleistocene is believed to have had positive effects on diversity as it supported the origin of new species while keeping extinction rates low [15, 18]. As a result, species were able to persist in montane or coastal refugia. The lack of gene flow caused by low dispersal capabilities led to high genetic differentiation within genera. As all Lentulidae are flightless, they are likely to have limited dispersal capabilities, as has been shown in the genus *Betiscoides* [9]. Consequently, this might also explain diversity patterns within the Lentulidae in general, even though the initial splits into several main clades or genera probably occurred much earlier. Several palaeorelictual insects of the Cape Floristic Region are known [5], suggesting that parts of the Cape Floristic Region may have served as refugia for quite a long time. Some represent ancient Gondwanan lineages. Some Lentulidae genera (*Betisocoides, Devylderia*) seem to be adapted to plants of typical fynbos plants, which radiated 70 my ago. The phylogeny of Orthoptera indicates that Lentulidae started radiating during that period as well [20]. It is thus unlikely that Lentulidae include ancient Gondwanan genera despite their high endemicity.

## Conclusions

Our results provide first insights in the phylogeny of the grasshopper family Lentulidae, indicating that the current subdivision into two subfamilies needs to be revised and that the East African *Usambilla* group represents a sister clade to the genera *Lentula* and *Eremidium*. Furthermore, species diversity within the genera seems to be higher than the current taxonomy suggests. Further research is needed including a broader taxon sampling and sequencing of additional genes.

## Methods

### Study objects

Members of the family Lentulidae are flightless and lack tympana. Further morphological features, such as shape of head or antenna, vary within this family. Lentulidae occur in South and East Africa with the center of diversity situated in South Africa. In total, 70 % of the Lentulidae are endemic to South Africa. The family is grouped into two subfamilies Lentulinae and Shelforditinae, the latter one only occurring in South Africa. Lentulinae currently comprise 88 species within 25 genera, Shelforditinae consist of ten genera with 44 species. Genera such as *Leatettix* and *Uvarovidium* had been classified as Hemiacridinae (family Acrididae) for some time, but were assigned to the Shelforditinae by Ritchie (1982). The East African *Usambilla* group belongs to the subfamily Lentulinae.

### Sampling

**Fig. 2:**
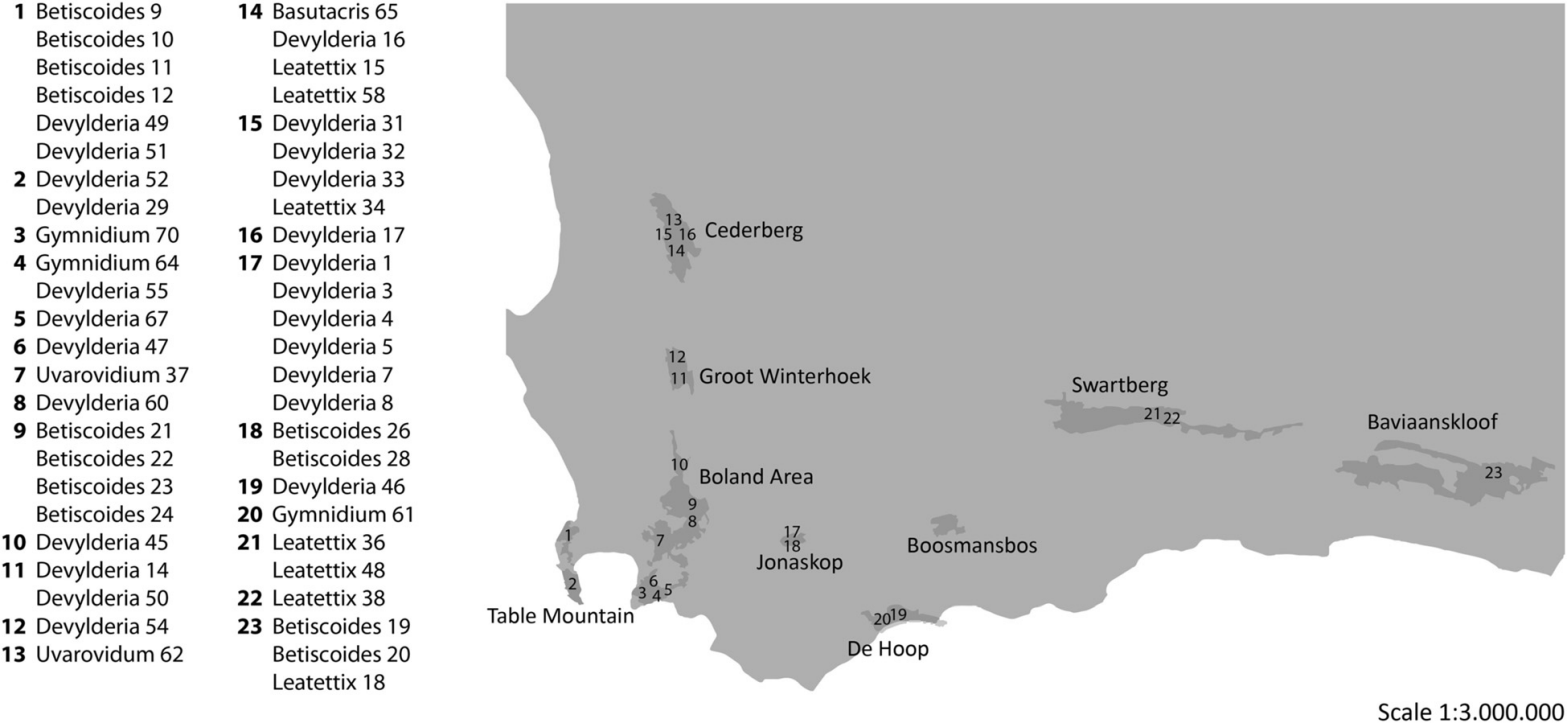
Shows the respective collection sites

A total of 50 Lentulidae specimens from six genera (*Basutacris*, *Betiscoides*, *Devylderia*, *Gymnidium*, *Leatettix* and *Uvarovidium*) were collected during four field trips from February 2012 to December 2013. Most specimens were collected in the eight reserves forming the UNESCO World Heritage Site “Cape Floral Region Protected Areas” and some in a private nature reserve “Jonaskop”. If possible, 3–5 specimens of each species were collected at each locality. Specimens were caught by sweep net or by hand and killed in a freezer. Afterwards they were stored in 99 % ethanol p.a. or dried and pinned. Information on specimens and localities and GenBank reference numbers is given in Fig. 2 and Table 3. Specimens are stored at the State Museum of Natural History in Stuttgart. *Sphingonotus rubescens* (Acrididae: Oedipodinae), *Frontifissia elegans* and *F. laevata* (Acrididae; Catantopinae) were chosen as outgroups.

**Table 3:** Information on specimen, locality (coordinates and reserve) and collection site. The site numbers are included in Fig. 2

Specimen	Site	Reserve	Coordinates	Genbank 12S	Genbank NDS
*Uvarovidium peninsulare* 37	7	Jonkershoek	S33 58.005 E18 55.195	KU206353	KU214623
*Uvarovidum peninsulare* 62	13	Cederberg	S32 25.341 E19 07.709	KU206364	KU214634
*Leatettix cf emota* 15	14	Cederberg	S32 25.917 E19 11.052	KU206335	KU214605
*Leatettix cf emota* 34	15	Cederberg	S32 25.704 E19 11.010	KU206351	KU214621
*Leatettix cf emota* 58	14	Cederberg	S32 25.917 E19 11.052	KU206362	KU214632
*Leatettix moraki* 36	21	Swartberg	S33 21.698 E22 05.094	KU206352	KU214622
*Leatettix moraki* 48	21	Swartberg	S33 21.698 E22 05.094	KU206356	KU214626
*Leatettix moraki* 38	22	Swartberg	S33 20.777 E21 58.702	KU234555	n/a
*Leatettix moraki* 18	23	Baviaanskloof	S33 38.085 E24 28.425	KU206338	KU214608
*Devylderia capensis* 1	17	Jonaskop	S33 58.163 E19 30.300	KU206324	KU214594
*Devylderia capensis* 5	17	Jonaskop	S33 58.163 E19 30.300	KU206327	KU214597
*Devylderia capensis* 8	17	Jonaskop	S33 58.163 E19 30.300	KU206329	KU214599
*Devylderia capensis* 7	17	Jonaskop	S33 58.163 E19 30.300	KU206328	KU214598
*Devylderia capensis* 3	17	Jonaskop	S33 58.163 E19 30.300	KU206325	KU214595
*Devylderia capensis* 4	17	Jonaskop	S33 58.163 E19 30.300	KU206326	KU214596
*Devylderia capensis* 14	11	Groot Winterhoek	S33 00.197 E19 04.348	KU206334	KU214604
*Devylderia* spec. 60	8	Hottentots Holland	S34 04.454 E19 03.702	KU206363	KU214633
*Devylderia capensis* 47	6	Kogelberg	S34 17.765 E19 07.470	KU234556	n/a
*Devylderia* spec. 55	4	Kogelberg	S34 21.355 E18 51.543	KU234557	n/a
*Devylderia capensis* 29	2	Table Mountain	S33 57.536 E18 23.268	KU206347	KU214617
*Devylderia coryphistoides* 49	1	Table Mountain	S34 19.038 E18 25.211	KU206357	KU214627
*Devylderia capensis* 51	1	Table Mountain	S34 19.038 E18 25.211	KU206359	KU214629
*Devylderia capensis* 45	10	Limietberg	S33 41.272 E19 05.750	KU206354	KU214624
*Devylderia capensis* 52	2	Table Mountain	S33 57.536 E18 23.268	KU206360	KU214630
*Devylderia capensis* 67	5	Kogelberg	S34 19.604 E18 50.224	KU234559	n/a
*Devylderia bothai* 46	19	De Hoop	S34 26.644 E20 25.301	KU206355	KU214625
*Devylderia coryphistoides* 17	16	Cederberg	S32 26.168 E19 10.929	KU206337	KU214607
*Devylderia coryphistoides* 31	15	Cederberg	S32 25.704 E19 11.010	KU206348	KU214618
*Devylderia capensis* 32	15	Cederberg	S32 25.704 E19 11.010	KU206349	KU214619
*Devylderia cf capensis* 33	15	Cederberg	S32 25.704 E19 11.010	KU206350	KU214620
*Devylderia* spec*.* 50	11	Groot Winterhoek	S33 00.197 E19 04.348	KU206358	KU214628
*Devylderia* spec*.* 54	12	Groot Winterhoek	S32 59.589 E19 03.548	KU206361	KU214631
*Devylderia bothai* 16	14	Cederberg	S32 25.917 E19 11.052	KU206336	KU214606
*Betiscoides meridionalis* 9	1	Table Mountain	S34 19.038 E18 25.211	KU206330	KU214600
*Betiscoides meridionalis* 10	1	Table Mountain	S34 19.038 E18 25.211	KU206331	KU214601
*Betiscoides meridionalis* 12	1	Table Mountain	S34 19.038 E18 25.211	KU206333	KU214603
*Betiscoides meridionalis* 11	1	Table Mountain	S34 19.038 E18 25.211	KU206332	KU214602
*Betiscoides meridionalis* 21	9	Hottentots Holland	S33 58.832 E19 07.903	KU206341	KU214611
*Betiscoides meridionalis* 22	9	Hottentots Holland	S33 58.832 E19 07.903	KU206342	KU214612
*Betiscoides meridionalis* 23	9	Hottentots Holland	S33 58.832 E19 07.903	KU206343	KU214613
*Betiscoides meridionalis* 24	9	Hottentots Holland	S33 58.832 E19 07.903	KU206344	KU214614
*Betiscoides meridionalis* 26	18	Jonaskop	S33 57.622 E19 31.168	KU206345	KU214615
*Betiscoides meridionalis* 28	18	Jonaskop	S33 57.622 E19 31.168	KU206346	KU214616
*Betiscoides meridionalis* 19	23	Baviaanskloof	S33 38.085 E24 28.425	KU206339	KU214609
*Betiscoides meridionalis* 20	23	Baviaanskloof	S33 38.085 E24 28.425	KU206340	KU214610
*Basutacris* spec*.* 65	14	Cederberg	S32 25.917 E19 11.052	KU206366	KU214636
*Gymnidium turbinatum* 61	20	De Hoop	S34 28.320 E20 27.120	KU234558	n/a
*Gymnidium turbinatum* 64	4	Kogelberg	S34 21.355 E18 51.543	KU206365	KU214635
*Gymnidium turbinatum* 70	3	Kogelberg	S34 19.528 E18 50.763	KU206367	KU214637

Sequences for 12S from the genera *Eremidium nr equuleus* (AY569277.1), *Lentula callani* (NC_020774.1) *Lentula obtusa* (AY569276.1), *Usambilla sagonai* (AY569279.1) and *Rhainopomma montanum* (Z97601.1) were obtained from Genbank for an extended data set for 12S. The data sets for 12S and NDS were concatenated, data for NDS was coded missing when sequences were not available (Table 3).

### DNA analysis

DNA was isolated from the insects’ hind leg muscles using the Qiagen DNeasy Blood and Tissue Kit (Qiagen GmbH, Hilden, Germany). We amplified two mitochondrial gene fragments: 12S rRNA and the gene fragment NDS (a combination of 16S rRNA, t-Leu and NADH-Dehydrogenase subunit 1 (ND1); see Table 4 for primer sequences). The gene fragment 12S was chosen as it typically amplifies well also in old samples (i.e. the pinned specimens used), whereas NDS was used because it is more variable and typically shows a better resolution than 12S. Two different polymerases were used for PCR reactions (HotMasterMix by 5Prime and HotStarTaq Master Mix by Qiagen). The reactions for both polymerases were compiled as follows: 26 μl of diH_2_O, 20 μl of HotStarMasterMix or HotStarTaq Master Mix respectively, 0.3 pmol of each primer and 50 ng of DNA template (total Vol. = 50 μl). HotStarMasterMix was used for amplifying 12S under the following conditions: 94 °C for 2 min., 37 Cycles of denaturation at 94 °C for 30 s., annealing at 45 °C for 30 s and elongation at 65 °C for 1 min and a final elongation step for 10 min at 65 °C. NDS was amplified with the HotStarTaq Master Mix under the following conditions: Initial denaturation at 96 °C for 20 min., 33 cycles of denaturation at 96 °C, annealing at 45 °C for and elongation at 68 °C for 1.30 min each following a final elongation step at 65 °C for 3 min. PCR products were visualized on an 1 % agarose gel stained with SYBR Green I (Biozym, Hessisch, Oldendorf, Germany). The products were purified using Roche High Pure PCR Product Purification Kit (Roche Deutschland Holding GmbH, Germany) and sequenced afterwards at MacroGen Cooperation (Amsterdam, The Netherlands) or on a MEGABACE 1000 automated sequencer at the University of Trier with the DYEnamic ET Terminator Cycle Sequencing Premixkit (GE Healthcare, Munich, Germany).

**Table 4:** Primer information for analyzed gene fragments 12S, 16S, t-Leu and ND1

Name	Gene	Sequence (5'-3')	Reference
12sai	12S rRNA	AAA CTA GGA TTA GAT ACC CTA TTA T	[27]
12sbi	12S rRNA	AAG AGC GAC GGG CGA TGT GT	[27]
NDII	16S rRNA	ACA TGA TCT GAG TTC AAA CCG G	[28]
NDS	ND1	TAG AAT TAG AAG ATC AAC CAG C	[29]

### Sequence analysis

Sequences were inspected in MEGA 6.0 [21] and aligned using ClustalW. The Gap opening penalty was set to 15 for pairwise and multiple alignments respectively and the gap extension penalty was set to 6.66 for both pairwise and multiple alignment. IUB was chosen as DNA Weight Matrix with a transition weight of 0.5. Delay Divergent cutoff was set to 30 %. The genetic distance (p-Distance) between the genera was calculated only for the 12S sequences, as NDS was missing in GenBank for the genera *Lentula*, *Eremidium*, *Rhainopomma* and *Usambilla*. For tree reconstruction with Bayesian Inference we first used PartitionFinder in order to detect possible partitions in the data set and to find the best-fitting substitution models applicable in MrBayes [22]. Subsets were defined before Partitionfinder was run by dividing NDS in the components 16S (including t-Leu) and ND1 as well as the coding gene ND1 into its coding positions. In total, five different partitions with different substitution models were calculated. The model GTR + I + G was the best-fitting model for 12S, 16S and first coding position of ND1; HKY + I was calculated for the second coding position of ND1 and HKY + G for the third coding position of ND1.

We performed a Bayesian analyses on the datasets using MrBayes v.3.1.2 [23, 24]. The analysis was run for 20 million generations, sampling trees every 2000 generations. The first 2500 trees were discarded before a consensus tree was calculated and visualized in FIGTREE v. 1.4.2 [25].

The Maximum Likelihood tree reconstruction was run in MEGA 6.0. In addition to PartitionFinder, we applied the goodness-of-fit test for each nucleotide model in order to find the best substitution model applicable in MEGA [26]. Based on the BIC (Bayesian Information Criterion) and AICc (corrected Akaike Information Criterion), we chose the best-fitting models. The best fitting model for the Maximum likelihood analysis was GTR + G (BIC: 6153.091). The Bootstrap method was chosen as phylogenetic test and the number of replications of bootstraps was set to 500. Gaps or missing data were treated with partial deletion with a cutoff of 95 %. The heuristic method was set to Nearest Neighbor Interchange with the first tree being calculated automatically. The Branch swap filter was set to very strong.

## Additional file

Additional file 1: Figure S1. Phylogeny of the analyzed genera belonging to Lentulidae. Maximum likelihood tree for the genes 12S and NDS. *Frontifissia* and *Sphingonotus* were defined as outgroups. (JPG 1183 kb)
