# Supplementary figures and images for: A preliminary phylogeny of the South African Lentulidae
Source: Hereditas. 2016 Jan 15;153:1. doi: 10.1186/s41065-015-0005-6 (PMC5224585; doi:10.1186/s41065-015-0005-6)

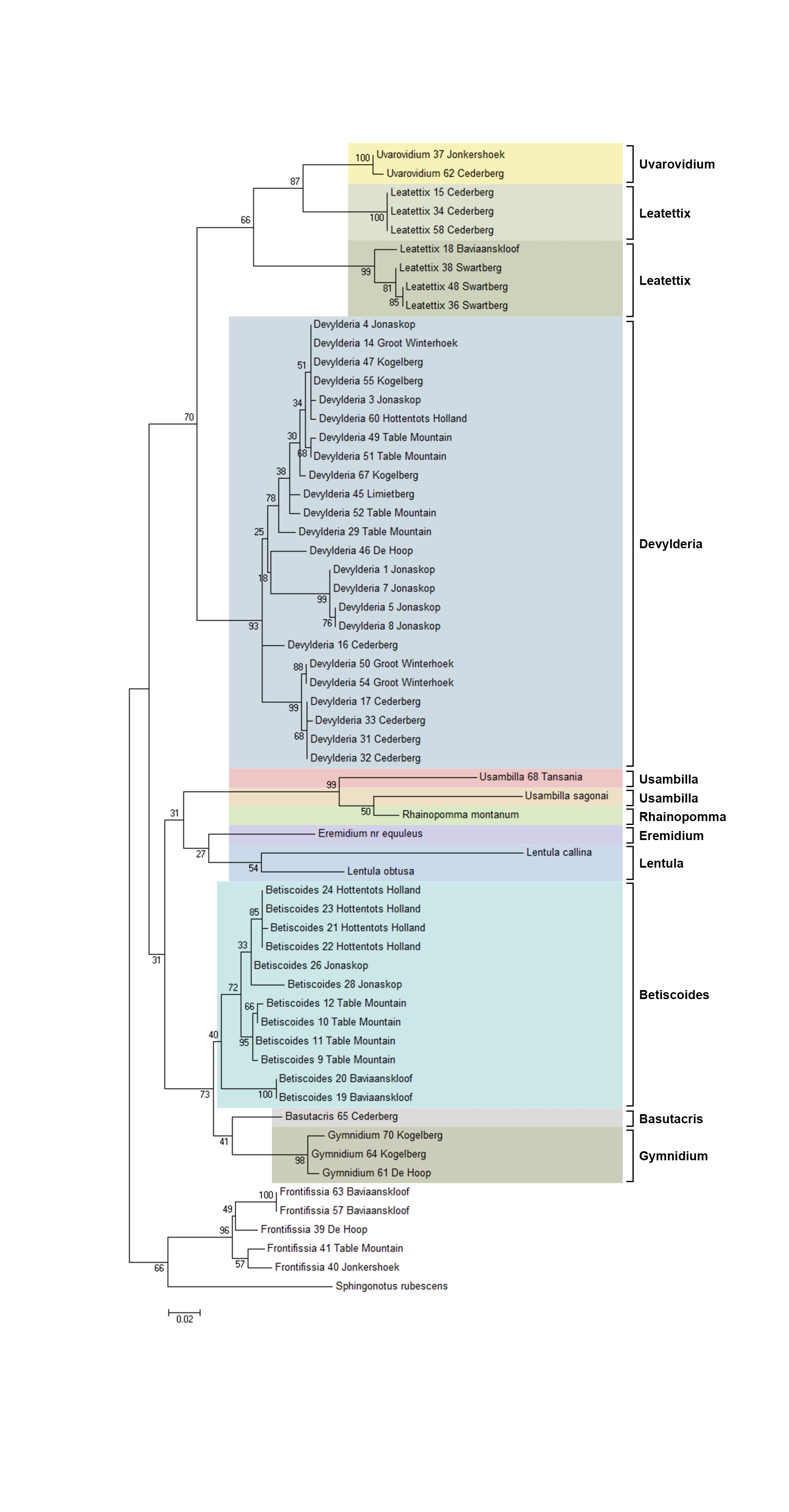

Supplement: Additional file 1: Figure S1. — Phylogeny of the analyzed genera belonging to Lentulidae. Maximum likelihood tree for the genes 12S and NDS. Frontifissia and Sphingonotus were defined as outgroups. (JPG 1183 kb) [file 41065_2015_5_MOESM1_ESM.jpg]
